# CD44v8-10 as a potential theranostic biomarker for targeting disseminated cancer cells in advanced gastric cancer

**DOI:** 10.1038/s41598-017-05247-7

**Published:** 2017-07-10

**Authors:** Eun-Seok Choi, Hyunjin Kim, Hyung-Pyo Kim, Yongdoo Choi, Sung-Ho Goh

**Affiliations:** 1 Precision Medicine Branch, Research Institute, National Cancer Center, 323 Ilsanro, Goyang, Gyeonggi-do 10408 Republic of Korea; 2 Molecular imaging & therapy branch, Research Institute, National Cancer Center, 323 Ilsanro, Goyang, Gyeonggi-do 10408 Republic of Korea; 3 Department of Environmental Medical Biology, Institute of Tropical Medicine, Yonsei University College of Medicine, Seoul, 03722 Republic of Korea

## Abstract

Gastric cancer is the third most common cause of cancer mortality, and the survival rate of stage IV advanced gastric cancer (AGC) patients with distant metastasis is very low. Thus, the detection and eradication of disseminated cancer cells by targeting cell surface molecules in AGC would improve patient survival. The hyaluronic acid receptor, CD44, has various isoforms generated by alternative splicing, and some isoforms are known to be correlated to gastric cancer. In this study, to find out the most appropriate CD44v for targeting AGC, we analysed the expression differences of CD44 isoforms at the mRNA level in stomach cancer cell lines as well as in 74 patients with AGC by using exon-specific qRT-PCR. Among the CD44v isoforms, CD44v8-10 was determined as the most promising biomarker for the development of theranostic agents of gastric cancer. Next, we synthesised the conjugate of anti-CD44v9 antibody with near-infrared fluorophore or photosensitiser, and then demonstrated its feasibility for target cell-specific imaging and photoimmunotherapy in gastric cancer. As a result, these conjugates have clearly demarcated the surface of CD44v8-10 expressing cancer cells and showed efficient phototoxic effects. Therefore, this study revealed that CD44v8-10 is the efficient theranostic biomarker to target disseminated cancer cells in AGC.

## Introduction

Stomach cancer is the third leading cause of cancer death in both sexes worldwide (723,000 deaths, 8.8% of the total). In particular, the highest estimated mortality rates are in Eastern Asia (24 per 100,000 in men, 9.8 per 100,000 in women)^1^. Thus, it is necessary to develop a therapeutic strategy to overcome the high mortality rates caused by stomach cancer. Stomach cancer is composed of heterogeneous cell populations that manifest malignancy by aberrantly regulating cell proliferation, differentiation, angiogenesis, migration, and metastasis^2^, and its carcinogenic process is complex^3, 4^. To obtain a comprehensive understanding of the pathophysiological status of cancer at a molecular level, an enormous amount of genome data is being profiled and analysed worldwide, but the only approved biomarker of tumour response to targeted agents in advanced gastric cancer (AGC) is human epidermal growth receptor (HER)-2. Although trastuzumab is an approved targeted therapeutic drug for the subgroup of (HER)-2-positive AGC, based on the results of a ToGA trial (phase III trastuzumab for gastric cancer), the majority of patients did not respond in the first-line setting. Therefore, identification of new biomarkers for detection and therapy of gastric cancer is an ongoing clinical challenge.

The single-pass transmembrane glycoprotein CD44, which binds to hyaluronic acid, has also been recognized as one of the markers to fit the purpose. It is implicated in tumour cell invasion and metastasis^5^, as well as many physiological phenomena related to tumour formation including cell migration, invasion, and metastasis^6^. The CD44 gene has ten constant exons and ten variable exons (v1-v10) placed between exon 5 and 16, and multiple isoforms of CD44 molecules are generated via alternative mRNA splicing of the ten variable exons. While the standard isoform of CD44 (CD44s) is known to predominate in hematopoietic cells and normal epithelial cell subsets, many variant isoforms (CD44v) with the extended extracellular stalk region are more prevalent in epithelial carcinomas^7^. It was reported that the CD44v3,8-10 isoform was involved in the metastasis of breast cancer^8^ and colorectal adenomas^9^, and CD44v3 and CD44v6 for colorectal cancer^10^. Furthermore, CD44v6 and CD44v9 interact with CD95, the death receptor, interfering with death receptor signalling and inhibiting apoptosis^11^.

Although the roles of CD44v in cancer stem cells (CSCs) remain elusive, it was reported that CD44(+) gastric cancer cells have the stem cell properties of self-regeneration and the ability to form differentiated programs when compared with CD44(−) cells^12^. Thus, CD44 variants might also be considered as susceptible targets for cancer stem cells^13^; however, this has not yet been confirmed^2^. Relapse of gastric cancer has been debated in terms of stem cell-like properties of cells in the lesions, and CD44v9^14^ and CD44v8-10 isoforms^12, 15^ were suggested as predictive markers as well as molecules for targeting CSCs. In addition, CD44v8-10, which interacts with and thereby stabilizes xCT at the plasma membrane, was reported as the key component contributing to reactive oxygen species (ROS) defence through upregulation of the synthesis of reduced glutathione (GSH)^5, 16^.

In addition, based on the idea that the binding between CD44 and sodium hyaluronate is mediated by many different intra/extracellular signalling pathways and is related to cancer proliferation and tumorigenesis^17^, clinical trials of antibody-mediated therapeutics are developed. Recently, Birzele *et al*.^18^ reported a CD44 blocking antibody, RG7356, which binds a constant region of the CD44 molecule, and it showed relevance for CD44s-expressing tumour cells. However, the suggested CD44v isoforms have not been screened and validated among patients yet.

Here, we analysed the differential expression of CD44v at the mRNA level in patients with AGC and compared the results with the expression of standard CD44s. Based on the differences in CD44v profiles, we also investigated the CD44v8-10 isoform as a new therapeutic target for disseminated cancer cells in AGC. Finally, we showed that conjugates of anti-CD44v9 antibody with near-infrared fluorophore or photosensitiser may have potential for specific imaging and photoimmunotherapy of gastric cancer.

## Results

### Differential expression of CD44 alternative spliced variants in gastric cancer cell lines and gastric cancer patient tissues

**Figure 1:**
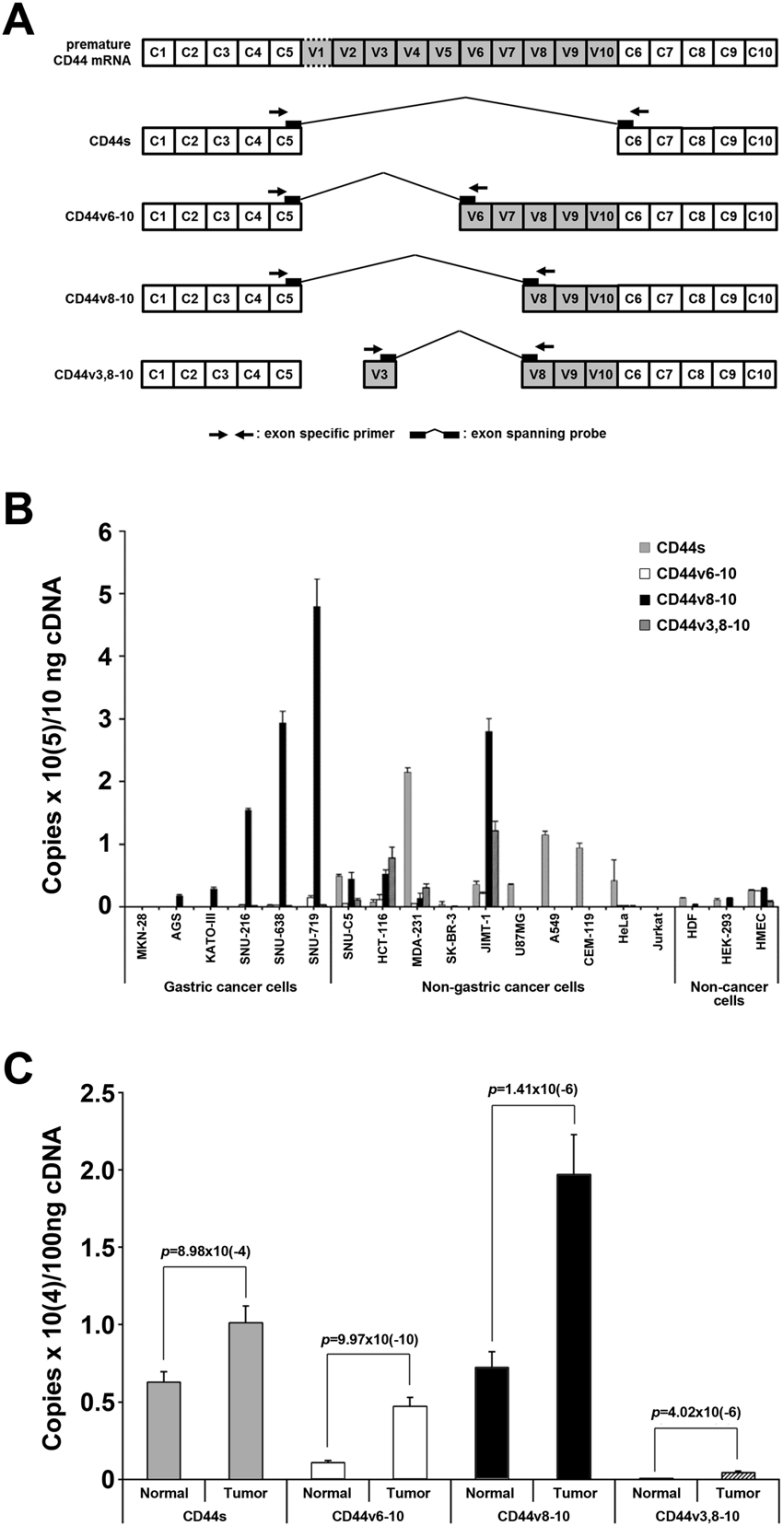
(**A**) Structure of human CD44 pre-mRNA and its alternative splice variants. To detect alternative splice variants, CD44s or CD44v specific TaqMan probes spanning the border of contiguous exons and primer pairs were applied. (**B**) The comparison of mRNA expression levels of CD44s and CD44v in the cell lines measured by absolute quantification of qRT-PCR. (**C**) The comparison of mRNA expression level of CD44s and CD44v between normal and tumour tissue of stomach cancer patients (*n* = 74).

From the previous study of profiling exon usage differences between normal and tumour tissues of 30 patients with early gastric cancer by using Affymetrix Exon 1.0ST array (GSE30727), we found CD44v to be a gene which shows significant differences in alternative splicing (*p*-value = 2.75 × 10^−3^) as well as in expression (*p* – value = 1.97 × 10^−4^) at the mRNA level. To validate this result in a separate sample set, we designed FAM-TAMRA TaqMan probes spanning the junction of alternative spliced exons and primer pairs specific for alternative splice variants of CD44s, CD44v6-10, CD44v8-10 and CD44v3,8-10 (Fig. 1A and Supplementary Table S1) as these are frequently reported splice variants in cancers.

First, we screened the variants using qRT-PCR in 19 cell lines and tissues from 74 patients with gastric cancer. The absolute quantification of four types of CD44 expression variants revealed that CD44v8-10 was the major isoform in gastric cancer cell lines except for MKN-28 (Fig. 1B). CD44s was the second most abundant species, but its expression was not prominent. The expression of CD44v6-10 or CD44v3,8-10 was also detected in gastric cancer cells but it was very low. In comparison, CD44s was the major isoform at the mRNA level in non-gastric cancer cell lines though some cell lines such as JIMT-1 showed CD44v8-10 as the major isoform (Fig. 1B). In non-cancer cell lines including HDF, HMEC, and HEK-293, the overall CD44 expression itself was much lower than that in cancer cell lines, and the level of alternative splice variant isoforms was not significantly higher than that of CD44s (Fig. 1B). When we analysed the CD44 variant expression in normal tumour-paired tissues from patients with gastric cancer (Table 1), CD44s and CD44v6-10 were detected in all normal and tumour tissues (*f* = 1.000), and CD44v8-10 was detected in 71 out of 74 normal tissues (*f* = 0.959) and 73 out of 74 tumour tissues (*f* = 0.986). In accordance with gastric cancer cell lines, CD44v8-10 was the most highly expressed form followed by CD44s (Fig. 1C). These results suggest that the CD44v8-10 splice variant is the major CD44 isoform in gastric cancer and its expression in tumours is much higher than that of other variants. Thus, CD44v8-10 can be used as a potential target for gastric cancer-specific therapies.

**Table 1:** Clinicopathological information of 74 advanced gastric cancer patients.

			No. of patients
Number of patients	Total		74
	Male		52
	Female		22
Age at diagnosis (yrs)	Range		21–86
	Mean ± SD		60.7 ± 13.3
Disease stage^†^	Tumor stage	T1a	0
		T1b	1
		T2	16
		T3	29
		T4a	22
		T4b	6
	Node stage	N0	26
		N1	11
		N2	13
		N3a	16
		N3b	8
	Metastasis stage	M0	63
		M1	11
Lauren type	Intestinal		33
	Diffuse		24
	Mixed		7
	Intermediate		8
	Not annotated		2
Borrmann type	Type I (protruded type)		1
	Type II (ulcerative type)		21
	Type III (ulceroinfiltrated type)		45
	Type IV (diffuse type)		6
	Not determined		1

^†^Stage classification follows the TNM classification system by International Union Against Cancer (UICC).

### Anti-CD44v9 antibody conjugated with near-infrared (NIR) fluorophore recognizes the CD44v8-10 expressing cells in tumours

**Figure 2:**
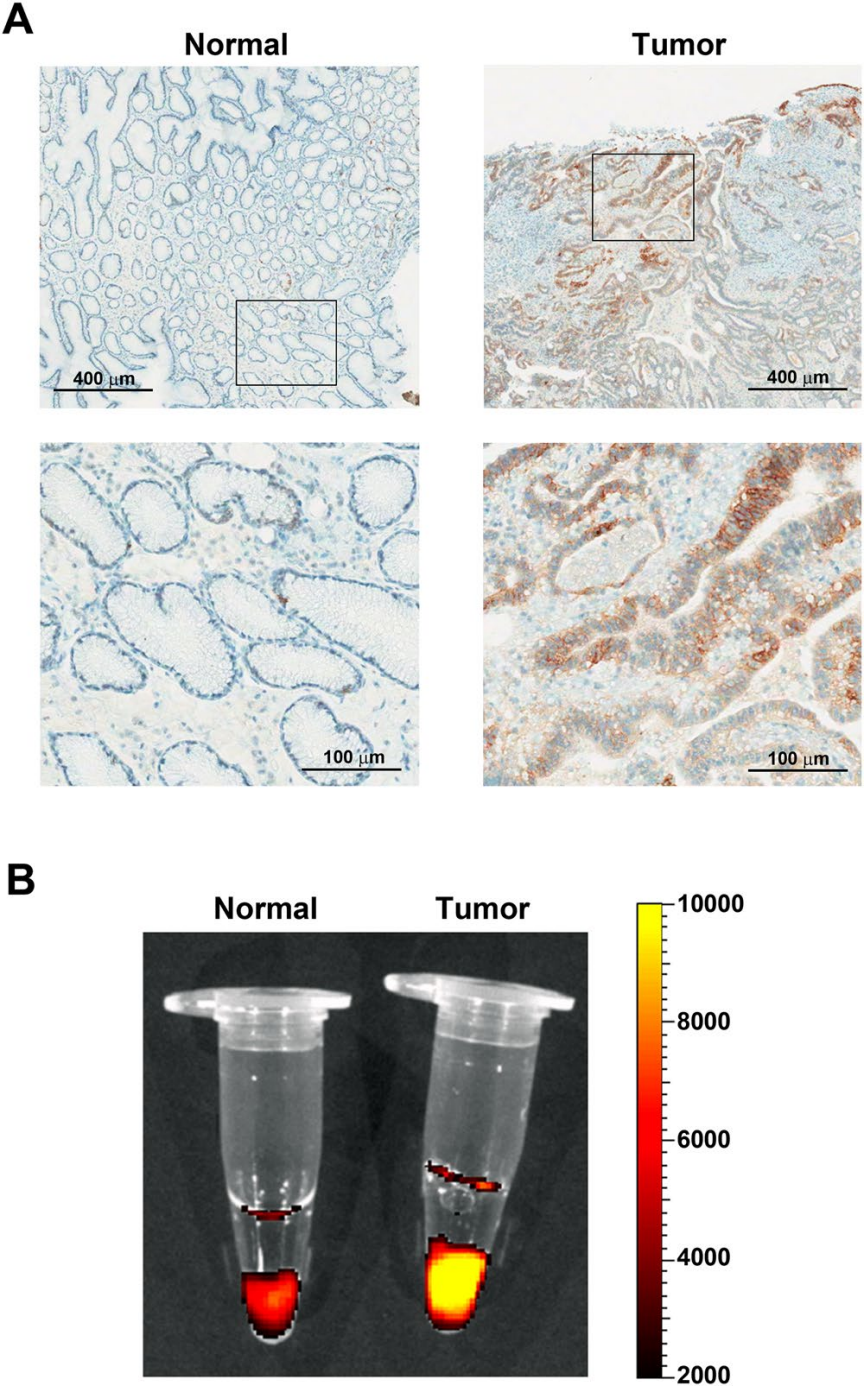
(**A**) Immunohistochemistry of normal-tumour paired gastric cancer tissue using anti-CD44v9 antibody. Normal tissue showed no CD44v9 signals in the section and has regularly arranged gastric ducts. In comparison, the CD44v9 signal is prevalent in tumour tissue and it has dysplastic phenotypes at the ductal regions. (**B**) Immunofluorescence stained images with tissue sample obtained from a gastric cancer patient using anti-CD44v9-ATTO680.

To confirm whether the protein level of CD44v8-10 is also elevated in tumour tissues, we performed immunohistochemistry using CD44 variable exon 9 specific antibody (RV3) on patients with high CD44v8-10 expression in tissue samples. In normal tissue, protein expression of the exon v8-10-containing CD44 variant was not detected except for specific regions such as endothelial cells of gastric glands. On the contrary, in tumour tissues, CD44v8-10 protein expression was observed in almost the entire region and was prominent in the region of dysplastic tissue (Fig. 2A). Therefore, it is suggested that the CD44v8-10 variant can be used to target gastric tumour tissues for imaging and therapy.

Next, we checked the feasibility of targeting the CD44v8-10 on fresh three-dimensional tissue samples using NIR fluorophore-conjugated anti-CD44v9 antibody. The same pairs of gastric cancer patient tissue samples that were used for immunohistochemistry were treated with ATTO680 fluorophore-conjugated anti-CD44v9 antibody, and the results were imaged with an *in vivo* imaging instrument (IVIS Lumina XR, Xenogen Corporation-Caliper, CA, USA, ex. 660/20, em. 710/40 nm). ATTO680 is a water-soluble NIR fluorophore and has a good fluorescence quantum yield in water (*Φ*_F_ = 0.19)^19^. Notably, the strongest fluorescence signal was observed in the tumour tissues as compared with that of normal tissues (Fig. 2B). These results suggest that NIR fluorophore-conjugated anti-CD44v9 antibody preferentially binds to the three-dimensional tumour mass expressing CD44v8-10 variant protein and therefore may be used for selective imaging of CD44v8-10-overexpressing gastric cancers.

### Establishment of the CD44v8-10 variant expressing cell line

To quantify the CD44 molecules on the cell surface, we performed immunocytochemistry of three gastric cancer cells (MKN-28, KATO-III, and SNU-638) and normal cells (HDF) to compare the protein expression with the expression level of mRNA. As expected, MKN-28 showed no signals on the membrane and the other three cell lines followed the expression patterns of mRNA. HDF and KATO-III have highly expressed CD44s and CD44v8-10, respectively, and SNU-638 expressed both CD44s and CD44v8-10 (Supplementary Fig. 1).

**Figure 3:**
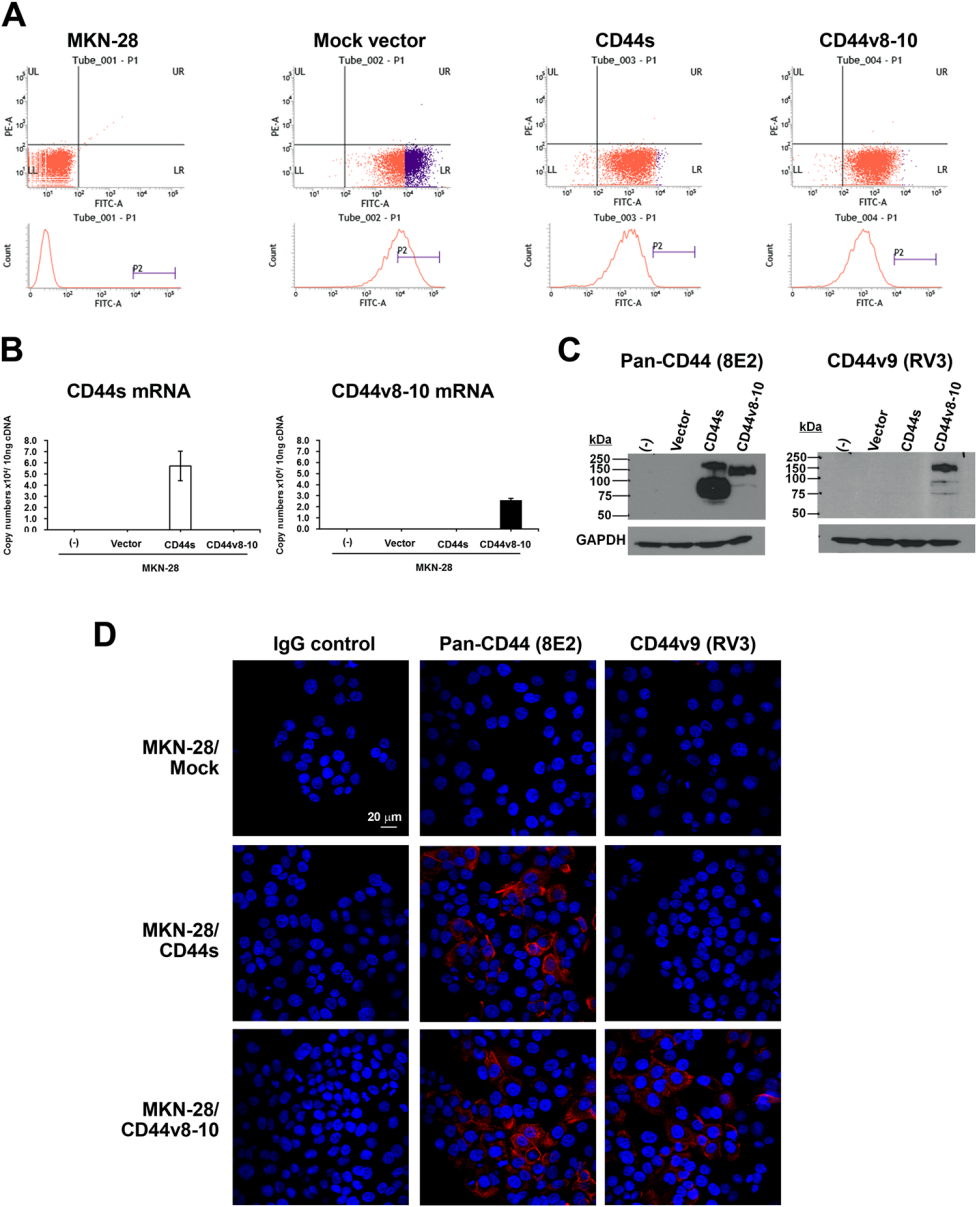
Establishment of stably CD44s and CD44v8-10expressing cells. (**A**) Stable expression of GFP-tagging open reading frame was confirmed by flow cytometry. (**B**) Expression of the stably expressed CD44s and CD44v8-10 was confirmed at mRNA level by qRT-PCR. (**C**) Protein level was confirmed by western blot using pan-CD44 antibody and CD44v9 antibody. (**D**) The location of CD44s and CD44v8-10 on MKN-28 cells was also confirmed by immunocytochemistry.

To test the efficacy of antibody-based targeting CD44v8-10, we set up cell lines that express only CD44s or CD44v8-10 by using the MKN-28 cell line. This line does not express CD44 due to hyper-methylation of the CD44 promoter region^20^. We first verified the expression of GFP-tagged CD44 variants by flow cytometry (Fig. 3A). MKN-28 was negative for fluorescence and all the other stable cell lines harbouring GFP or GFP-tagged CD44 showed green fluorescence. To confirm the expression of different CD44 variants, we quantified absolute mRNA expression by qRT-PCR. Measuring the levels of CD44s or CD44v8-10 variant in each stable cell line showed that each cell line expressed its own CD44 species exclusively (Fig. 3B). The expression of CD44 proteins in each cell line was confirmed by western blot. As expected, pan-CD44 antibody detects both CD44s and CD44v8-10, but anti-CD44v9 antibody only detects CD44v8-10 in stable cell lines (Fig. 3C). Finally, we observed the expression of CD44 variants on the membranes of the stable cell lines via an immunocytochemistry assay (Fig. 3D). Western blotting revealed that the pan-CD44 antibody detects both CD44s and CD44v8-10 expression in MKN-28 cells, but anti-CD44v9 antibody detects only cells that express CD44v8-10.

### Specificity of NIR fluorophore-conjugated anti-CD44v9 antibody for targeting CD44v8-10 expressing cells

It is often difficult to discriminate or detect tumour margins or disseminated peritoneal micrometastatic gastric tumours during a surgical operation. Therefore, development of target cell-specific imaging probes for gastric cancer cells is a current clinical challenge. To establish the CD44v8-10-specific diagnostics, we conjugated the anti-CD44v9 antibody with NIR fluorophore ATTO680 (CD44v9-ATTO680), and then checked whether this conjugate could specifically bind to gastric cancer cells with CD44v8-10 overexpression.

**Figure 4:**
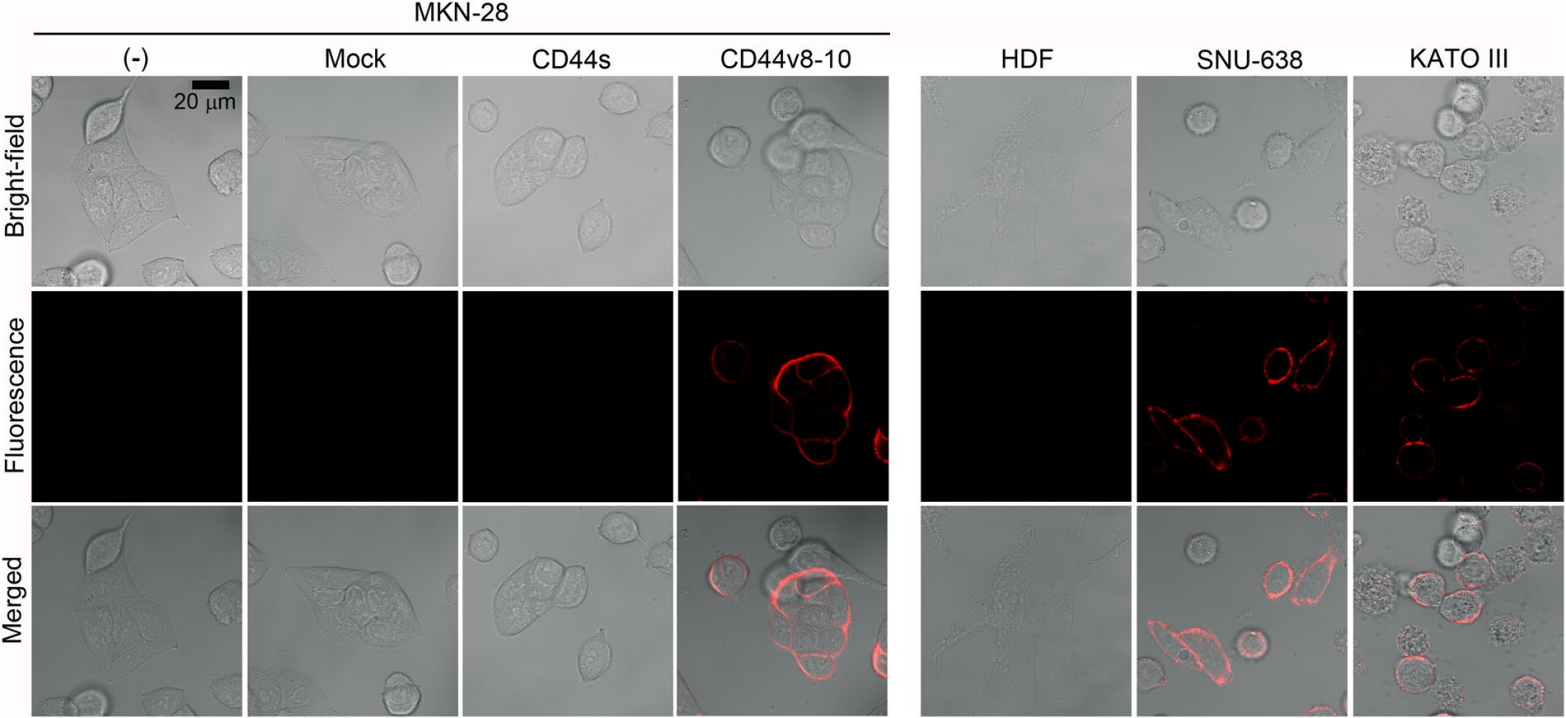
Specificity of ATTO680-conjugated anti-CD44v9 antibody on CD44v8-10(−) or (+) cells confirmed by NIR fluorescence image. No fluorescent signal was detected in MKN-28 cells, mock vector transfected MKN-28 cells, CD44s transfected MKN-28 cells, and HDF cells. The CD44v8-10 expressing MKN-28 cells, SNU-638 and KATOIII cells, clearly showed fluorescence on the lining of the cell membrane. Scale bar indicates 20 μm.

To verify the CD44v8-10-specific binding of the conjugated antibody on the cell surface, we treated CD44v9-ATTO680 to CD44(−) MKN-28, MKN-28 transfected with mock vector, CD44s expressing MKN-28, and CD44v8-10 variant stable MKN-28 cell lines as well as with CD44(−) HDF cells and two CD44v8-10(+) gastric cancer cells (KATO-III and SNU-638) (Fig. 4). As expected from the qRT-PCR results, MKN-28 cells without CD44v8-10 expression did not show any detectable fluorescence signal on the cell surface. We observed that the anti-CD44v9-ATTO680 conjugate recognises the cells with high expression of CD44v8-10 (KATO-III and SNU-638) and the CD44v8-10 stable MKN-28 cell line (Fig. 4).

### Targeted photoimmunotherapy of CD44v8-10 expressing cells by using anti-CD44v9-photosensitiser conjugate

**Figure 5:**
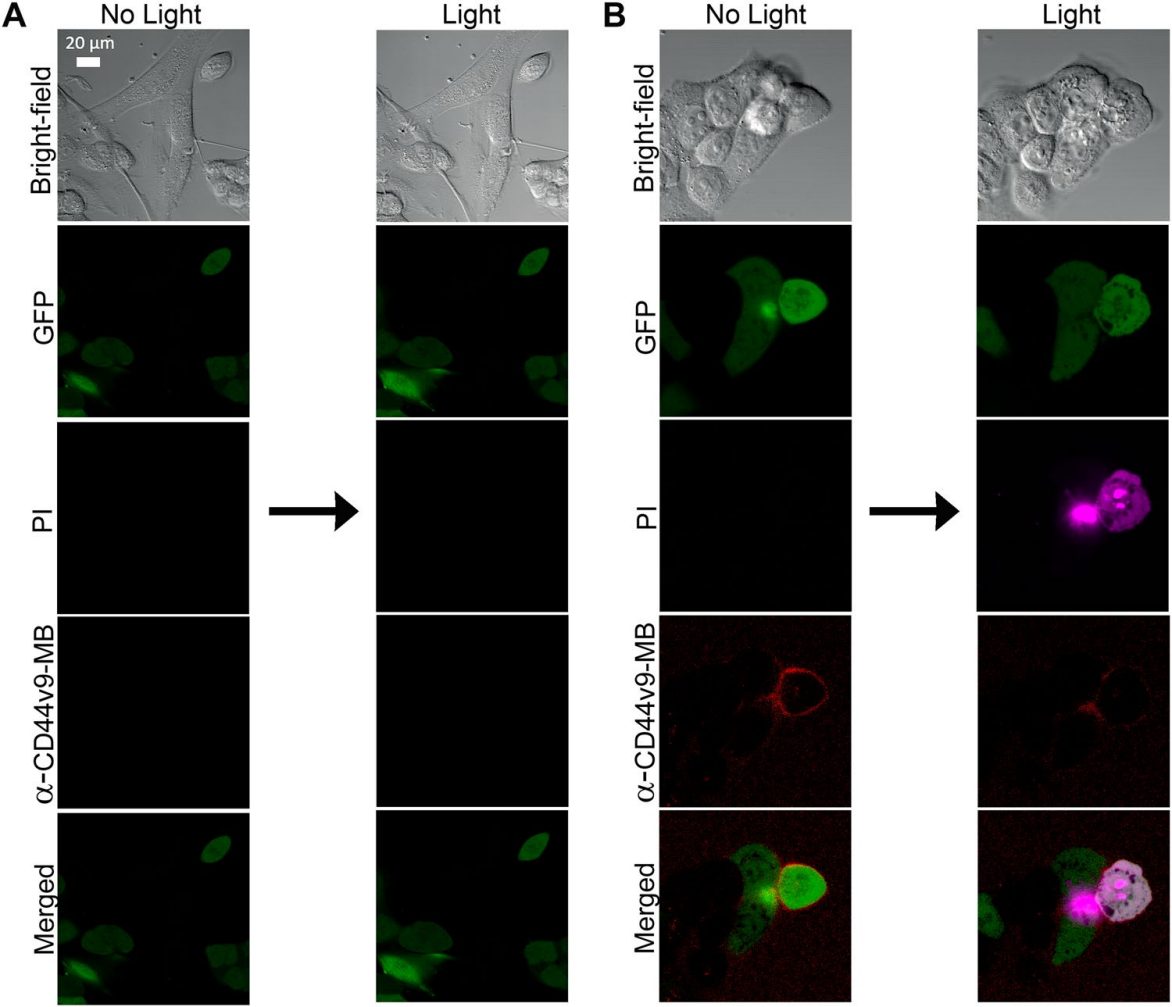
Targeted photoimmunotherapy of CD44v8-10(+) cancer cells using anti-CD44v9-MB conjugate. Results obtained with CD44(−) HDF/CD44(−) Mock MKN-28 cell combination (**A**) and CD44(−) MKN-28/CD44v8-10(+) MKN-28 cell combination (**B**). Cells were treated with anti-CD44v9-MB conjugate (8.96 μg/0.3 mL, 0.2 μM MB equivalent) for 1 h, and then the cells were irradiated with 633-nm laser light. Scale bar indicates 20 μm.

Methylene blue (MB), an FDA-approved NIR fluorophore, has long been used for NIR fluorescence imaging in clinics. In addition, its use in photodynamic therapy (PDT) of cancers has been exploited. That is, laser exposure to this dye causes not only NIR fluorescence (fluorescence quantum yield, *Φ*_F_ = 0.053)^21^ but also the highly efficient generation of singlet oxygen (singlet oxygen quantum yield, *Φ*_Δ_ = 0.49)^22^, thereby selectively killing cancer cells^23^. To examine whether the MB conjugated anti-CD44v9 antibody could specifically recognise only CD44v8-10 expressing cells among CD44v8-10(−) cells, we treated it in two combinations. The first combination was CD44(−) normal HDF and MKN-28 cells transfected with mock vector (Fig. 5A). The second combination was CD44(−) MKN-28 and CD44v8-10(+) MKN-28 cells (Fig. 5B). Cells were treated with propidium iodide (PI) before and after light irradiation to analyse damage to cell membranes. The results showed no detectable CD44v9-MB fluorescence or PI staining in the CD44(−) HDF/MKN-28 transfected with mock vector mixed condition before and after 633-nm laser irradiation (Fig. 5A). In contrast, Fig. 5B showed specific binding of CD44v9-MB conjugate to CD44v8-10(+) MKN-28 even when combined with CD44(−) MKN-28 cells, and subsequent light irradiation-induced selective cell damage of the conjugate-bound CD44v8-10(+) MKN-28 cells. These results suggest the applicability of anti-CD44v9-MB antibody conjugate for NIR fluorescence detection and photoimmunotherapy of both primary gastric cancer cells and disseminated cancer stem cell-like CD44v8-10(+) cells in the peritoneal cavity in AGC.

## Discussion

The survival prospects of many patients with AGC are not hopeful because most of them suffer from relapses caused by distant metastasis. The most common sites to which cancer cells spread are the liver, peritoneum, and distant lymph nodes. Among these distant metastases in AGC, peritoneal dissemination is more frequently associated with death from AGC (53–60%) than hepatic metastases (~40%)^24^. The 5-year survival rate with metachronous peritoneal carcinomatosis was reported as only 3%^25^. Therefore, it is imperative that adequate treatment is received to eradicate cancer cells seeded into the abdominal cavity in order to improve disease-free survival in patients with gastric cancer. Recently, a few intraperitoneal immunotherapies using catumaxomab targeting epithelial cell adhesion molecule (EpCAM) antigen combined with cytoreductive surgery (CRS) or hyperthermic intraperitoneal chemotherapy (HIPEC) were reported to significantly increase the survival of patients^26, 27^. However, targeting a more cancer cell-specific antigen was not applied. Thus, it is crucial to understand the pathophysiological states of stomach cancer at a molecular level to develop targeted therapeutics as well as identify biomarkers for the diagnosis of distant metastasis. Notably, an enormous amount of data from The Cancer Atlas Genome (TCGA) project is being generated and analysed to reveal targeting opportunities^28^. Many biomarkers of gastric cancer have been identified and therapeutic biomarkers are being used as potential targets of cancer therapy, but lack accurate clinical evidences because the range of molecular characteristics of gastric cancer is wide depend on studies (7–44%)^29^.

In this study, we focused on the CD44 molecule, the hyaluronic acid receptor that has several valuable clinical significances. It is known that CD44, especially CD44v isoforms, is highly expressed in cancers, and the expression of CD44v isoforms is differential among cancer species^30^. Recently, CD44 has been recognized as a cell surface marker for cancer initiating cells^16, 31^, and correlations between isoform and cancer stem cells were reported in breast cancer^32^, prostate cancer^33^, colorectal cancer^34^, and primary lung cancer^35^. In gastric cancer, relapse of gastric cancer has been debated in terms of stem cell-like properties of cells in the lesions and CD44v8-10 has been reported as the cancer stem cell marker in gastric cancer^15^. It was reported that CD44 has the ability to cause peritoneal dissemination in gastric cancer^36^. Therefore, according to these reports, CD44v is a promising therapeutic target to eliminate the peritoneal disseminated cancer stem cells in gastric cancer. In this study, we confirmed CD44v8-10 is the major isoform of the CD44 variants in AGC (Fig. 1). The anti-CD44v9 antibody used in this study could detect CD44v6-10 and CD44v3,8-10 as well, because the v9 exon is also included in these variants. However, as shown in Fig. 1B and C, the expression level of CD44v8-10 mRNA is considerably higher than that of the other two variants in both gastric cancer cell lines and tumour tissues. In particular, CD44v3,8-10 is rarely expressed. These differences would imply that CD44v8-10 is predominantly detected rather than CD44v6-10 or CD44v3,8-10. The expression of CD44s in tumour tissue is prominent because human tissue does not consist of pure cancer cells, but also contains haematopoietic cells, fibroblasts, and stromal cells that express CD44s. As shown in Fig. S1, the fibroblast cell, HDF, shows no signal upon CD44v9 antibody treatment, but shows a high signal in response to the pan-CD44 antibody. This result coincides with the RT-PCR result shown in Fig. 1B. Therefore, targeting CD44v8-10 is considerably more efficient than targeting CD44s to eliminate cancer cells from a heterogeneous cell population. In addition, some M1 patients showed substantially higher levels of CD44v8-10/CD44s than M0 patients did. This is in contrast to the CD44v6-10/CD44s levels, which were similar in both the groups (Supplementary Fig. S3). Of note, the gastric cancer cell lines originating from metastasized region (SNU-216) or ascites (SNU-638) definitely also showed higher CD44v8-10 levels than the cell lines from primary sites (AGS) did. Thus, we can conclude that the conjugates of anti-CD44v9 antibody with NIR fluorophore (ATTO680) or photosensitizer (MB) have potential usefulness for targeted imaging or therapy of advanced gastric cancer.

Photoimmunotherapy is a new cancer treatment modality that combines cancer specific antibodies, photosensitisers, and NIR light to selectively kill target cancer cells. Recently Sato *et al*. developed photosensitiser-trastuzumab conjugates and showed their potential for fluorescence imaging and phototherapy in the disseminated peritoneal carcinomatosis animal model of HER2-overexpressing gastric cancer^37^. Thus, we examined the idea that photosensitiser-conjugated anti-CD44v9 antibody could be used as a theranostic agent for selective detection and therapy of gastric cancers. Therefore, we examined the significance of CD44v8-10 as a major target for disseminated gastric cancer. Based on the CD44(−) and CD44v8-10 expressing cell lines, we conducted controlled experiments to analyse the therapeutic efficacy of photosensitiser-conjugated anti-CD44v9 antibody. The CD44v8-10 targeting antibody was conjugated with methylene blue, which has been used in photodynamic therapy due to the generation of singlet oxygen when it absorbs NIR light^23^. We confirmed the anti-CD44v9-MB antibody specifically binds only to CD44v8-10-expressing cells, even when those cells are mixed with non-CD44v8-10(+) cells. It is worth noting that the cytotoxic effect was turned on only after laser exposure (Fig. 5). In addition, it is also noteworthy that, as shown in Figs 2 and 4, the NIR fluorophore (*i.e*., ATTO680)-conjugated anti-CD44v9 antibody showed its potential for specific NIR fluorescence detection of CD44v8-10 expressing cells in the *in vitro* cell and *ex vivo* tumour-tissue studies. Thus, it could be applied to image guided surgery to resect micro tumour masses that are difficult to discriminate with the naked eye. In addition, CD44v8-10 was reported as the key component contributing to ROS defence through upregulation of the synthesis of reduced glutathione (GSH)^5, 16^. Thus, selective targeting of CD44v8-10-positive cancer cells by PDT has a greater translational advantage than targeting a constant region of CD44, such as RG7356^18^, which is also expressed ubiquitously on stromal and fibroblast cells, although it is higher in cancer cells.

In this study, we analysed the differential expression of CD44v at the mRNA level in patients with AGC and compared this with the expression of standard CD44s. Based on differences in the CD44v profile, we demonstrated that the CD44v8-10 isoform is a potential therapeutic target using NIR fluorophore or photosensitiser-conjugated anti-CD44v9 antibody. Taken together, these results suggest that targeting the CD44v8-10 isoform will provide new therapeutic strategies to increase the disease-free survival rates of patients with AGC by eradicating metastasis.

## Methods

### Cell lines and patient stomach tissues

Cell lines were obtained from ATCC (USA) (MKN-28, AGS, KATOIII, HCT-116, MDA-231, SK-BR-3, JIMT-1, U87MG, A549, CEM-119, HeLa, Jurkat and HDF) and from Korean Cell Line Bank (Seoul National University, Korea) (SNU-216, SNU-638, SNU-719, and SNU-C5) were cultured with designated media supplemented with 10% fetal bovine serum and 1x penicillin-streptomycin at 37 °C 5% CO_2_ incubator. Human stomach cancer tissues and peri-tumoral normal counter parts were excised within 10 min from the gastrectomy, and preserved in RNAlater solution until RNA purification at 4 °C. All patients provided informed consent prior to collection of tissues, and all methods were performed in accordance with the relevant guidelines and regulations approved by the Institutional Review Board of National Cancer Center of Korea (NCCNCS13732).

### Preparation of total RNA and cDNA synthesis

Total RNA from patient tissue was extracted using Trizol reagent (Thermo Fisher Scientific, USA) and then purified with RNeasy column with DNaseI (Qiagen) digestion to remove residual genomic DNA. Two microgram of total RNA primed with poly-d(T)_18–20_ primer was synthesized to first strand cDNA using Transcriptor cDNA synthesis kit (Roche, Switzerland).

### Quantitative real-time Polymerase Chain Reaction

The oligonucleotide primers to planking CD44 variable exons and TaqMan probes that are anchor on the border of linked exons were designed (Fig. 1). 10 ng of cDNA used for each amplification of each CD44v with 5 pmoles of each forward and reverse primers and 10 pmoles of FAM-TAMRA labelled oligonucleotide primers (Bioneer, Korea), and 1x TaqMan master mix (Roche, Switzerland) in 10 ul reaction mixture. All the amplifications were carried out triplicate in LightCycler LC480 Realtime PCR system (Roche, Switzerland) with 45 cycles of thermal cycles of 94 °C for 10 sec and 60 °C for 30 sec. Results were analysed by Roche LC480 software v1.5 using absolute quantification by interpolation from the curves of standard from 1.0 × 10^2^ to 1.0 × 10^9^ copies/reaction and non-template control.

### CD44 variants expression detection

To detect the expression of pan-CD44 and CD44v8-10 variants at the protein level, anti CD44 antibody (8E2) (Cell Signaling Technology, USA) and anti-CD44v9 antibody (RV3) (Cosmobio, Japan) were used. Briefly, cell were cultured on coverslip were fixed with paraformaldehyde and incubated with 1:100 diluted antibody and then secondary antibody labelled with fluorescent dye. After washing with D-PBS, fluorescent images of the recorded by Axiovision Software (Carl Zeiss, Germany).

### Establishment of CD44 variant overexpression cell line

The open reading frames of CD44s and CD44v8-10 variants were amplified and cloned into GFP tagged pHRST lentiviral vector. These expression plasmids were propagated by transfecting HEK-293T cells, and harvesting lentiviral particle using Lenti-X concentrator (Clontech, Japan). CD44 negative MKN-28 gastric cancer cell lines were infected with each CD44s or CD44v8-10 expression lentivirus and stable transfectants were selected by GFP expression.

### Synthesis of anti-CD44v9 antibody conjugates and assessment of its theranostic efficiency

The antibody that recognized the extended stalk region containing variable exon 9 (RV3) was conjugated with N-hydroxysuccinimidyl (NHS)-functionalized ATTO680 (NHS-ATTO680; ATTO-TEC GmbH, Siegen, Germany) or MB (ATTO-MB2; ATTO-TEC GmbH, Siegen, Germany). In brief, anti-CD44v9 antibody (10 ug) was reacted with NHS-functionalized ATTO680 or MB (1 ug), in a phosphate buffered saline (PBS, 10 mM, pH 7.4) at room temperature for 1 h in a light protected place with gentle shaking. Byproducts including unreacted ATTO680 or MB were removed from the mixture using a Sephadex G25 gel filtration column (PD MiniTrap G-25, GE Healthcare, USA), and then the products were concentrated by Amicon Ultra-0.5 mL centrifugal filters (cut off: 50 kDa, EMD Millipore, USA). The resulting conjugate was kept at 4 °C before use.

Immunofluorescence images of patient tissues, anti-CD44v9-ATTO680 (0.1 μM ATTO680 equivalent) was diluted in 0.1 mL 1% BSA containing PBS buffer. Diluted anti-CD44v9-ATTO680 was added onto #68 patient stomach tissues sliced from frozen tissue and incubated for 1 h at R.T. After treatment, stained tissues were washed with 4 times and then optical images were obtained using Xenogen (ex. 660/20 nm, em. 710/40 nm, exposure time: 1 sec).

For detecting of CD44v8-10 in stable cell lines, MKN-28, mock vector transfected MKN-28, MKN-28/CD44s and HDF for negative controls and MKN-28/CD44v8-10, SNU-638, KATO III for positive controls were seeded at a density of 5 × 10^4^/well on a 4-well Lab-Tek II chambered cover glass (Thermo Fisher Scientific, USA) and incubated overnight for cell attachment. CD44v9-ATTO680 conjugates were diluted in the medium (1.96 μg/0.5 mL, 0.01 μM ATTO680 equivalent) and treated for 1 h. Fluorescence images were acquired using a confocal laser scanning microscope (Carl Zeiss LSM510 META, 40x, ex. 633 nm/ex. 679–754 nm).

*In vitro* photoimmunotherapy was performed in the mixed cell condition to evaluate whether anti-CD44v9-MB conjugate can selectively detect and damage the target cancer cells only. In the first set, CD44(−) HDF cells (5 × 10^3^ cells) was mixed with MKN-28 transfected mock vector (5 × 10^3^ cells) and seeded into 8-well Lab-Tek chamber. In the second set, CD44(−) MKN-28 and CD44v8-10(+) MKN-28 cells (5 × 10^3^ cells, respectively) were mixed and seeded into 8-well Lab-Tek chamber. After incubating the cells for 24 h for cell attachment, existing cell culture medium was replaced with fresh cell culture medium containing anti-CD44v9-MB conjugate (8.96 μg/0.3 mL, 0.2 μM MB equivalent), and the cells were treated for 1 h. Propidium iodide (PI, 3 μM) was co-treated to the cells to analyse damage of cell membranes. For photoimmunotherapy, the cells were irradiated with 633-nm laser for 7 min. The 633-nm He/Ne laser (5 mW) used in this test was the one equipped in confocal microscope to obtain fluorescence images in the light-irradiated region. Confocal fluorescence images were obtained using confocal microscopy (Carl Zeiss LSM 780, GFP: ex. 488 nm, em. 493–598 nm, PI: ex. 561 nm, em. 570–659 nm, MB: ex. 633 nm, em. 679–754 nm) before and after light irradiation.

### Statistical analyses

The statistical significance of differences between groups was determined using Student’s *t*-test (paired). *P*-values less than 0.05 were considered statistically significant.

## Electronic supplementary material

Supplementary Information

## References

[1] Ferlay J (2015). Cancer incidence and mortality worldwide: sources, methods and major patterns in GLOBOCAN 2012. Int J Cancer.

[2] Naor D, Nedvetzki S, Golan I, Melnik L, Faitelson Y (2002). CD44 in cancer. Crit Rev Clin Lab Sci.

[3] Wu K (2009). Molecular basis of therapeutic approaches to gastric cancer. J Gastroenterol Hepatol.

[4] Yin M, Hu Z, Tan D, Ajani JA, Wei Q (2009). Molecular epidemiology of genetic susceptibility to gastric cancer: focus on single nucleotide polymorphisms in gastric carcinogenesis. Am J Transl Res.

[5] Ishimoto T (2011). CD44 variant regulates redox status in cancer cells by stabilizing the xCT subunit of system xc(−) and thereby promotes tumor growth. Cancer Cell.

[6] Hiraga T, Ito S, Nakamura H (2013). Cancer stem-like cell marker CD44 promotes bone metastases by enhancing tumorigenicity, cell motility, and hyaluronan production. Cancer Res.

[7] Tanabe KK, Ellis LM, Saya H (1993). Expression of CD44R1 adhesion molecule in colon carcinomas and metastases. Lancet.

[8] Bourguignon LY (1998). CD44v(3,8-10) is involved in cytoskeleton-mediated tumor cell migration and matrix metalloproteinase (MMP-9) association in metastatic breast cancer cells. J Cell Physiol.

[9] Kopp R, Fichter M, Schalhorn G, Danescu J, Classen S (2009). Frequent expression of the high molecular, 673-bp CD44v3,v8-10 variant in colorectal adenomas and carcinomas. Int J Mol Med.

[10] Banky B (2012). Characteristics of CD44 alternative splice pattern in the course of human colorectal adenocarcinoma progression. Mol Cancer.

[11] Mielgo A, van Driel M, Bloem A, Landmann L, Gunthert U (2006). A novel antideatic mechanism based on interference of Fas signaling by CD44 variant isoforms. Cell Death Differ.

[12] Takaishi S (2009). Identification of gastric cancer stem cells using the cell surface marker CD44. Stem Cells.

[13] Klonisch T (2008). Cancer stem cell markers in common cancers - therapeutic implications. Trends Mol Med.

[14] Hirata K (2013). CD44 variant 9 expression in primary early gastric cancer as a predictive marker for recurrence. Br J Cancer.

[15] Lau WM (2014). CD44v8-10 is a cancer-specific marker for gastric cancer stem cells. Cancer Res.

[16] Nagano O, Okazaki S, Saya H (2013). Redox regulation in stem-like cancer cells by CD44 variant isoforms. Oncogene.

[17] Misra S (2011). Hyaluronan-CD44 interactions as potential targets for cancer therapy. FEBS J.

[18] Birzele F (2015). CD44 Isoform Status Predicts Response to Treatment with Anti-CD44 Antibody in Cancer Patients. Clin Cancer Res.

[19] Lee SF, Vérolet Q, Fürstenberg A (2013). Improved super-resolution microscopy with oxazine fluorophores in heavy water. Angew Chem Int Ed.

[20] Sato S, Yokozaki H, Yasui W, Nikai H, Tahara E (1999). Silencing of the CD44 gene by CpG methylation in a human gastric carcinoma cell line. Jpn J Cancer Res.

[21] Matsui A (2010). Real-time near-infrared fluorescence-guided identification of the ureters using methylene blue. Surgery.

[22] Fernandez JM, Bilgin MD, Grossweiner LI (1997). Singlet oxygen generation by photodynamic agents. J Photochem Photobiol B.

[23] Tardivo JP (2005). Methylene blue in photodynamic therapy: From basic mechanisms to clinical applications. Photodiagnosis Photodyn Ther.

[24] Okines A (2010). Gastric cancer: ESMO Clinical Practice Guidelines for diagnosis, treatment and follow-up. Ann Oncol.

[25] Baiocchi GL (2014). Follow-up after gastrectomy for cancer: results of an international web round table. World J Gastroenterol.

[26] Goere D (2014). Treatment of gastric peritoneal carcinomatosis by combining complete surgical resection of lesions and intraperitoneal immunotherapy using catumaxomab. BMC Cancer.

[27] Heiss MM (2010). The trifunctional antibody catumaxomab for the treatment of malignant ascites due to epithelial cancer: Results of a prospective randomized phase II/III trial. Int J Cancer.

[28] Rubio-Perez C (2015). In silico prescription of anticancer drugs to cohorts of 28 tumor types reveals targeting opportunities. Cancer Cell.

[29] Baniak N, Senger JL, Ahmed S, Kanthan SC, Kanthan R (2016). Gastric biomarkers: a global review. World J Surg Oncol.

[30] Yan Y, Zuo X, Wei D (2015). Concise Review: Emerging Role of CD44 in Cancer Stem Cells: A Promising Biomarker and Therapeutic Target. Stem Cells Transl Med.

[31] Zoller M (2011). CD44: can a cancer-initiating cell profit from an abundantly expressed molecule?. Nat Rev Cancer.

[32] Al-Hajj M, Wicha MS, Benito-Hernandez A, Morrison SJ, Clarke MF (2003). Prospective identification of tumorigenic breast cancer cells. Proc Natl Acad Sci USA.

[33] Collins AT, Berry PA, Hyde C, Stower MJ, Maitland NJ (2005). Prospective identification of tumorigenic prostate cancer stem cells. Cancer Res.

[34] Dalerba P (2007). Phenotypic characterization of human colorectal cancer stem cells. Proc Natl Acad Sci USA.

[35] Wang P (2013). Identification and characterization of cells with cancer stem cell properties in human primary lung cancer cell lines. PLoS One.

[36] Nishimura S, Chung YS, Yashiro M, Inoue T, Sowa M (1996). CD44H plays an important role in peritoneal dissemination of scirrhous gastric cancer cells. Jpn J Cancer Res.

[37] Sato K, Choyke PL, Kobayashi H (2014). Photoimmunotherapy of gastric cancer peritoneal carcinomatosis in a mouse model. PLoS One.

