# Supplementary material for: CD44v8-10 as a potential theranostic biomarker for targeting disseminated cancer cells in advanced gastric cancer
Source: Sci Rep. 2017 Jul 10;7:4930. doi: 10.1038/s41598-017-05247-7 (PMC5503939; doi:10.1038/s41598-017-05247-7)
Supplement: Supplementary file 1 — Supplementary Information [file 41598_2017_5247_MOESM1_ESM.pdf]

CD44v8-10 as a potential theranostic biomarker for targeting disseminated cancer cells in advanced gastric cancer

Authors: Eun-Seok Choi[1,3,†], Hyunjin Kim[2,†], Hyung-Pyo Kim[3], Yongdoo Choi[1,2,*], Sung-Ho Goh[1,*]

†These two authors equally contributed to this study.

Corresponding author: Sung-Ho Goh & Yongdoo Choi

Affiliations: [1]Precision Medicine Branch and [2] Molecular imaging & therapy branch, Research Institute, National Cancer Center, 323 Ilsanro, Goyang, Gyeonggi-do, 10408, Republic of Korea, [3]Department of Environmental Medical Biology, Institute of Tropical Medicine, Yonsei University College of Medicine, Seoul 03722, Republic of Korea.

Contact information:

Phone: +82-31-920-2477

e-mail: andrea@ncc.re.kr /ydchoi@ncc.re.kr

Address: 323 Ilsan-ro, Goyang, Gyeonggi-do, Republic of Korea, 10408

Supplementary Table S1. Sequences of primers and Taqman probes for quantitative RT-PCR.

Target	Forward primer (5′ – 3′)<br>Reverse primer (5′ - 3′)<br>Probe sequences (5′FAM – TAMRA3′)	Amplicon size	Annealing Temp (°C)
CD44s	CTT TTC TAC TGT ACA CCC CAT C<br>GTG AGT GTC CAT CTG ATT CAG<br>CTG CTA CCA GAG ACC AAG ACA CAT TCC	149 bp	54
CD44v6-10	CAT CCC AGA CGA AGA CAG TC<br>TCT TCC GTT GTA CTA CTA GGA GT<br>CAG AAT CCC TGC TAC CAT CCA GGC A	92 bp	54
CD44v8-10	CAT CCC AGA CGA AGA CAG TC<br>GCT GAA GCG TTA TAC TAT GAC TG<br>CAG AAT CCC TGC TAC CAA TAT GGA CTC	90 bp	54
CD44v3,8-10	GGG AGC CAA ATG AAG AAA AT<br>GGA TTT GCA GTA GGC TGA AG<br>ATC TCC AGC ACC AAT ATG GAC TCC AGT	142 bp	54

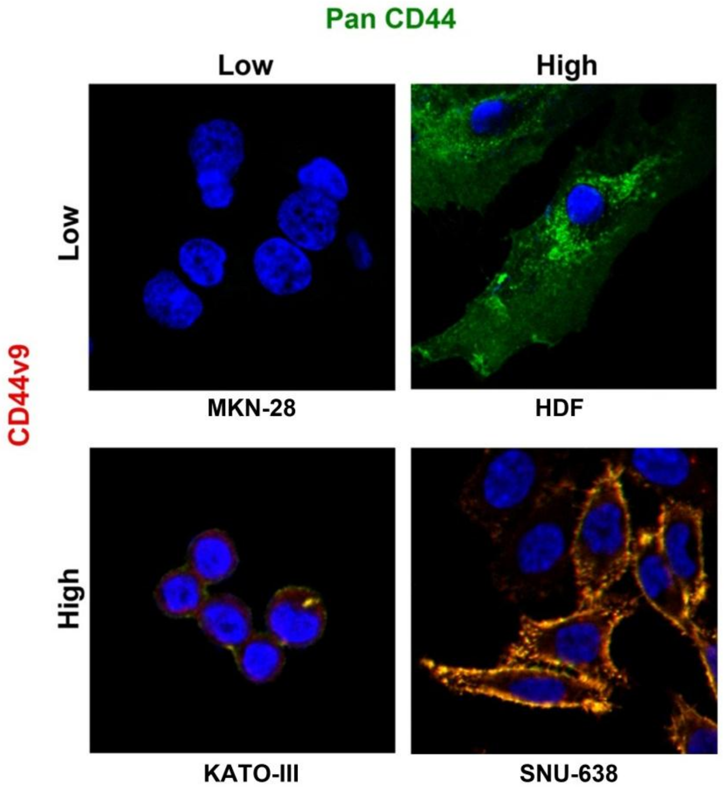

Supplementary Figure S1. Immunocytochemical staining of CD44s and CD44v8-10 molecules on cell lines using anti-pan-CD44 (8E2) and anti-CD44v9 (RV3) antibodies. The expression in MKN-28 stomach cancer cell line shows no positive signal. KATO-III cells showed very low level of CD44s but highly expressed CD44v8-10. SNU-638 cells showed both CD44s and CD44v8-10 at high level. Non-cancer cell line HDF showed only CD44s expression.

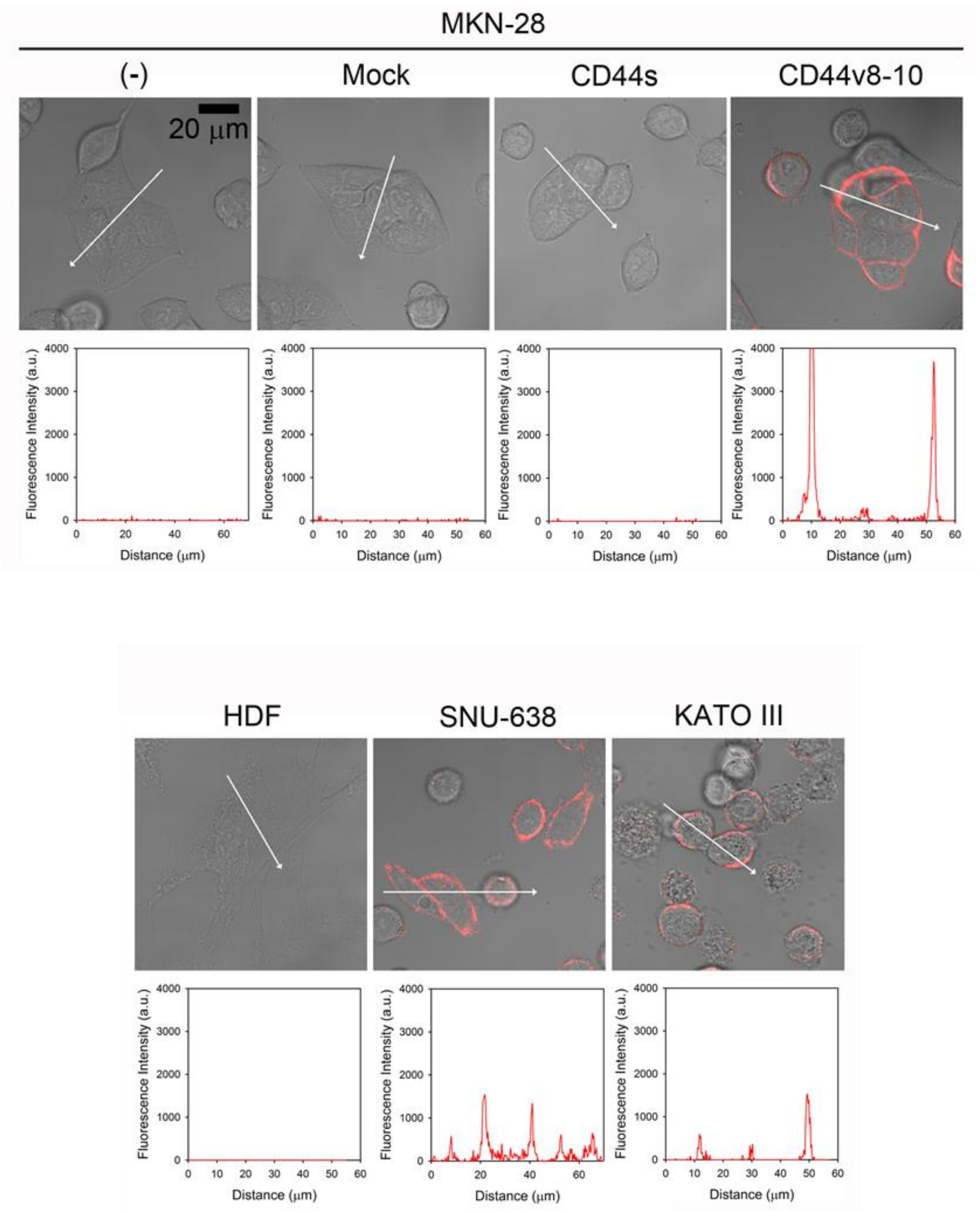

Supplementary Figure S2. Quantitative analysis for binding with CD44v9 in different cell lines. Fluorescence intensity across the area indicated by white arrows was analyzed using ZEN software.

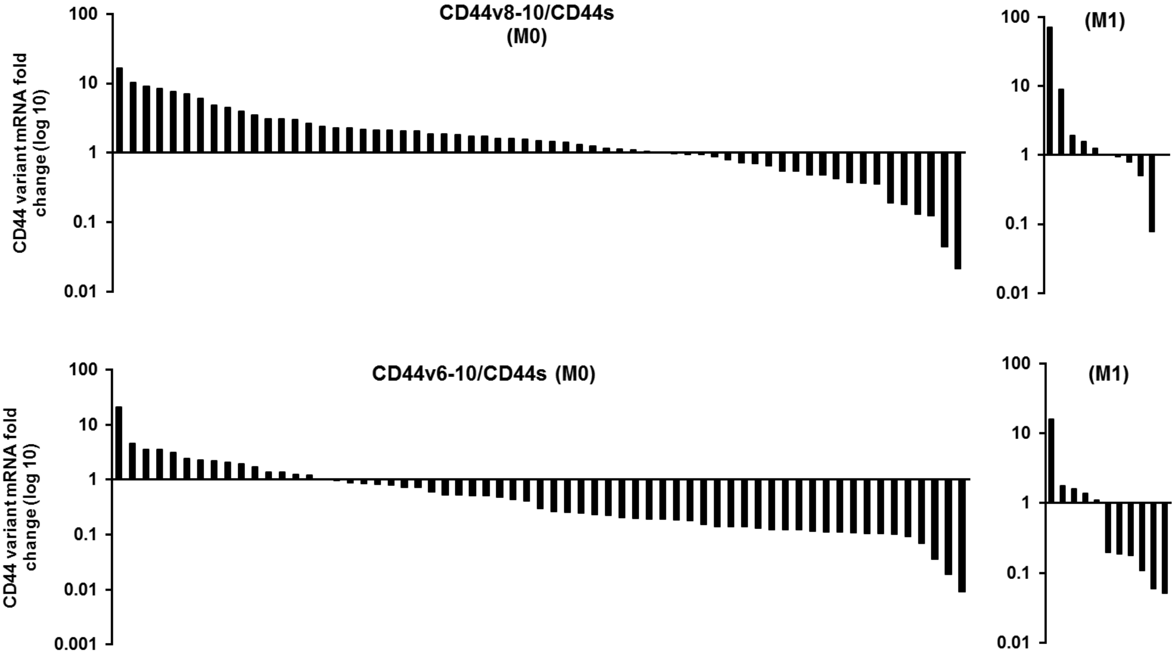

Supplementary Figure S3. Ratio of CD44v8-10/CD44s (upper panels) and CD44v6-10/CD44S (lower panels) according to metastasis stage (M0: w/o metastasis, M1: with distant metastasis) in advanced gastric cancer patients (*n*=74). CD44v8-10/CD44S ratio is higher in more patients in M0 group. In addition, M1 patient showed higher CD44v8-10/CD44S ratio than CD44v6-10/CD44S.
